# Structural characterization of CYP144A1 – a cytochrome P450 enzyme expressed from alternative transcripts in *Mycobacterium tuberculosis*

**DOI:** 10.1038/srep26628

**Published:** 2016-05-26

**Authors:** Jude Chenge, Madeline E. Kavanagh, Max D. Driscoll, Kirsty J. McLean, Douglas B. Young, Teresa Cortes, Dijana Matak-Vinkovic, Colin W. Levy, Stephen E. J. Rigby, David Leys, Chris Abell, Andrew W. Munro

**Affiliations:** 1 Manchester Institute of Biotechnology, Centre for Synthetic Biology of Fine and Specialty Chemicals (SYNBIOCHEM), Faculty of Life Sciences, The University of Manchester, Manchester M1 7DN, United Kingdom; 2 Department of Chemistry, University of Cambridge, Lensfield Road, Cambridge CB2 1EW, United Kingdom; 3 Centre for Molecular Microbiology and Infection, Imperial College London, London, United Kingdom; 4 Department of Pathogen Molecular Biology, Faculty of Infectious and Tropical Diseases, London School of Hygiene and Tropical Medicine, Keppel Street, London WC1E 7HT, United Kingdom

## Abstract

*Mycobacterium tuberculosis* (*Mtb*) causes the disease tuberculosis (TB). The virulent *Mtb* H37Rv strain encodes 20 cytochrome P450 (CYP) enzymes, many of which are implicated in *Mtb* survival and pathogenicity in the human host. Bioinformatics analysis revealed that CYP144A1 is retained exclusively within the *Mycobacterium* genus, particularly in species causing human and animal disease. Transcriptomic annotation revealed two possible CYP144A1 start codons, leading to expression of (i) a “full-length” 434 amino acid version (CYP144A1-FLV) and (ii) a “truncated” 404 amino acid version (CYP144A1-TRV). Computational analysis predicted that the extended N-terminal region of CYP144A1-FLV is largely unstructured. CYP144A1 FLV and TRV forms were purified in heme-bound states. Mass spectrometry confirmed production of intact, His_6_-tagged forms of CYP144A1-FLV and -TRV, with EPR demonstrating cysteine thiolate coordination of heme iron in both cases. Hydrodynamic analysis indicated that both CYP144A1 forms are monomeric. CYP144A1-TRV was crystallized and the first structure of a CYP144 family P450 protein determined. CYP144A1-TRV has an open structure primed for substrate binding, with a large active site cavity. Our data provide the first evidence that *Mtb* produces two different forms of CYP144A1 from alternative transcripts, with CYP144A1-TRV generated from a leaderless transcript lacking a 5′-untranslated region and Shine-Dalgarno ribosome binding site.

*Mycobacterium tuberculosis* (*Mtb*) is the causative agent for tuberculosis (TB), a chronic, infectious human disease that is responsible for the death of more than 1.5 million people annually. Major efforts have been made in recent years to produce new, effective antibiotics against *Mtb*^1^,^2^. New anti-TB drugs are desperately needed as a result of the emergence of drug-, multidrug- and extensively drug-resistant strains of *Mtb*, which have arisen as a consequence of factors including poor patient compliance with drug regimens. The “deadly synergy” between *Mtb* and the human immune deficiency virus (HIV) has also been an important factor contributing to the spread of TB and increase in human morbidity^3^. The best studied form of *Mtb* is H37Rv^4^, a virulent strain which has 20 genes encoding cytochrome P450 enzymes (P450s or CYPs)^1^,^4^,^5^. P450 monooxygenase enzymes are hemoproteins that typically catalyse the activation of molecular oxygen and the insertion of an atom of oxygen into a substrate bound close to the heme iron^6^. The high proportion of the *Mtb* genome dedicated to P450s implicates these enzymes in multiple important biochemical functions, and the anticipation that *Mtb* P450s may be involved in the pathogenicity and survival of the bacterium in the host has led to a focus of drug discovery efforts on *Mtb* P450s for new anti-TB compounds^7^,^8^,^9^. Characterization of P450 enzymes from *Mtb* has provided evidence for their involvement in the metabolism of lipids and sterol molecules, the oxidative modification of respiratory menaquinone and the production of cyclic dipeptide secondary metabolites^1^,^7^,^10^,^11^,^12^,^13^,^14^.

Currently, six of the *Mtb* P450s have been crystallized and structures of these enzymes have been determined in both ligand-free and various substrate/inhibitor-bound forms^7^,^9^,^11^,^13^,^15^,^16^,^17^. Physiological roles have been identified for CYP142A1 and CYP125A1 as host cholesterol catabolizing enzymes^17^,^18^, and also for CYP124A1 in sterol and/or branched chain fatty acid oxidation^11^. CYP132A1 was also proposed to be involved in fatty acid metabolism based on its similarity to eukaryotic CYP4 family fatty acid hydroxylases^19^. CYP51B1 is related to eukaryotic sterol 14α-demethylases (with fungal CYP51 enzymes being important targets for azole class drugs), while CYP121A1 is involved in secondary metabolite synthesis, catalysing the oxidative crosslinking of the aromatic side chains of the cyclic dipeptide cyclo-*L*-Tyr-*L*-Tyr (cYY) to form the product mycocyclosin. The *CYP121A1* gene was reported to be essential for *Mtb* viability, although the function of mycocyclosin remains unclear^13^,^15^,^20^. In view of the important roles demonstrated for the aforementioned P450 enzymes in *Mtb* viability and pathogenicity, it is important to investigate the physiological roles of the remaining P450s and to explore their potential involvement in bacterial physiology and survival in the host.

The identification of the first prokaryotic sterol demethylase (CYP51B1) in *Mtb* led to the proposal that azole antifungal drugs, which are effective, clinically-used inhibitors of fungal sterol demethylases, could be used as novel drugs for treating TB. The crystal structure of CYP51B1 was determined in complex with the antifungal drug fluconazole, revealing direct ligation of the P450 heme iron by a nitrogen atom from one of the inhibitor’s triazole rings. Although fluconazole is not very effective against *Mtb*^15^, the azole antifungals econazole and clotrimazole were found to be active against both latent and multidrug-resistant strains of *Mtb* in murine model systems^8^,^21^. However, the broad spectrum activity of these azole compounds has limited their application as systemic therapeutics. In addition to the potential of azole drugs as novel therapeutics against *Mtb*, they are also useful tools for probing the structure and function of the *Mtb* P450s^8^. Recently, *Mtb* CYP144A1, encoded by the H37Rv gene *Rv1777*^22^,^23^, was identified to bind tightly to several azole antifungals, raising the possibility that this P450 could be a target for novel anti-TB drug development.

In this paper, structural, biochemical, transcriptomic and bioinformatics data are presented to provide a detailed characterization of CYP144A1 and its potential as a *Mtb* drug target. The evolutionary ancestry of CYP144A1 is explored, and its conservation across the *Mycobacterium* genus is consistent with its importance to *Mtb*. Data from transcriptome annotation are reported, which reveal that alternative transcripts of CYP144A1 are produced, leading to the production of different forms of the P450 protein. The expression and purification of these different forms of CYP144A1 are described, as is the comparative analysis of their spectroscopic and ligand binding properties. Furthermore, the successful crystallization of the shorter form of CYP144A1 (CYP144A1-TRV) and the first X-ray crystal structure of a CYP144A1 protein are reported, enabling novel insights into this enzyme, its active site organization and its relationship to other *Mtb* P450 enzymes.

## Results and Discussion

### Bioinformatics and evolutionary studies of CYP144A1

**Figure 1:**
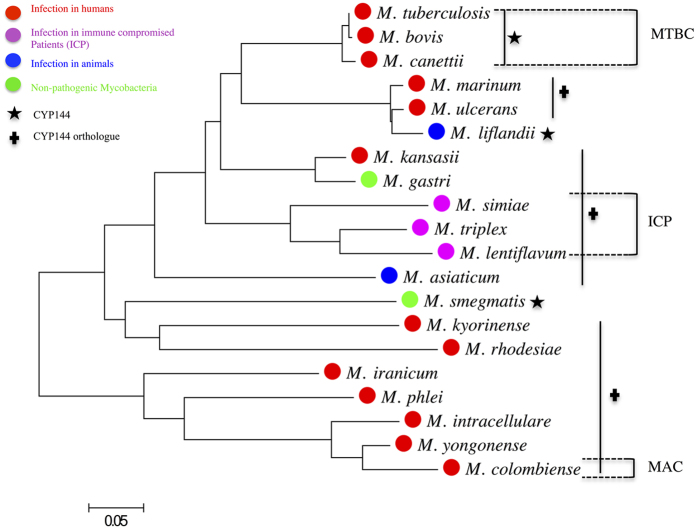
Phylogenetic tree illustrating the evolutionary relationship of CYP144A1 to other mycobacterial CYP144 family P450s. Protein sequence alignment of CYP144A1 was made with CYP144 sequences from other mycobacteria. Protein sequence conservation between the CYP144 proteins in all species ranges from 58–100%, with higher conservation of CYP144A1 sequence identity within the *Mtb* complex (MTBC), and the lowest conservation in the *M. avium* complex (MAC). The 0.05 reference scale marks a 5% estimated sequence variance. The figure was generated using MEGA6^44^.

The *Mtb* gene *Rv1777* (*CYP144A1*) from the pathogenic strain H37Rv (which encodes the cytochrome P450 enzyme CYP144A1) is located in a region of the *Mtb* genome rich in genes encoding proteins of unknown function. Thus, the prediction of the physiological function of CYP144A1 has been difficult from its genetic context alone^22^. The nearby gene *Rv1771* encodes a *L*-gulono-1,4-lactone dehydrogenase shown to catalyse the final step required in *L*-ascorbic acid biosynthesis^24^, while the *Rv1781c* gene is predicted to encode a glucanotransferase enzyme (MalQ or amylomaltase)^25^,^26^. However, ligand-binding experiments with CYP144A1 using various sugars did not result in P450 heme spectral shifts that would be consistent with substrate-like binding (data not shown). The *eccB5* and *eccC5* genes (*Rv1782* and *Rv1783*) encode predicted components of the ESX-5 protein export system, related to the better known ESX-1 virulence factor secretion system that exports the proteins ESX-1 (ESAT-6) and ESX-2 (CFP-10) into the host^27^. A little further downstream of *CYP144A1* are the *Rv1785c* and *Rv1786* genes, which encode the cytochrome P450 CYP143A1 and a likely 3Fe-4S ferredoxin redox partner for the P450. A BLAST search using the *Mtb* CYP144A1 protein sequence revealed that CYP144 P450s are highly conserved within *Mtb* strains (~99–100% identity), and also amongst the closely related *Mtb* complex (MTBC) members, which includes *M. bovis* (99%) and *M. canettii* (99%). Apparent orthologues of CYP144A1 occur in other pathogenic mycobacterial species, such as in *M. marinum* (80%) *and M. ulcerans* (79%), though typically with lower sequence identity to CYP144A1. The majority of species containing CYP144A1 orthologues are directly associated with a human disease, including *M. colombiense* (64% identity to *Mtb* CYP144A1), a member of the MAC (*Mycobacterium avium* complex) which can infect HIV patients with low CD4 cell counts^28^,^29^,^30^,^31^. CYP144A1 orthologues are also encoded by other mycobacteria isolated from patients with compromised immune systems (e.g. *M. simiae*, 76%; *M. triplex*, 80%; and *M. lentiflavum*, 76%). Among other CYP144A1-related sequences are CYP144A4 from the amphibian pathogen *M. liflandii* (77%), CYP144 orthologues from *M. asiaticum* (73%) which infects primates, and from the bacteria *M. smegmatis* (66%) and *M. gastri* (82%) which are typically considered to be non-pathogenic (Fig. 1).

No orthologues of CYP144 were observed outside the *Mycobacterium* genus to date. The apparent exclusive conservation of CYP144 P450 family enzymes in the mycobacteria suggests the retention of an important catalytic role. In previous studies, the *CYP144A1* gene was deleted from the *Mtb* H37Rv genome to investigate its effect on the growth and viability. It was found that the *Mtb CYP144A1* deletion strain was viable and grew *in vitro*, but that the growth rate of the deletion strain was substantially lower than that of the wild-type *Mtb* H37Rv. In addition, the *CYP144A1* deletion strain was markedly more susceptible to growth inhibition by azole drugs than the wild-type *Mtb*^22^. These data point to important function(s) for CYP144A1 in *Mtb*, and possibly to its participation in the *Mtb* stress response mechanisms. Consistent with this conclusion is the finding that *CYP144A1* is among the mycobacterial genes induced following bacterial growth arrest with vancomycin^32^. Vancomycin inhibits *Mtb* cell wall synthesis, and thus a potential function for this P450 may be related to the stress response to cell wall damage.

### Identification of a truncated form of the CYP144A1 P450 from transcriptome analysis

**Figure 2:**
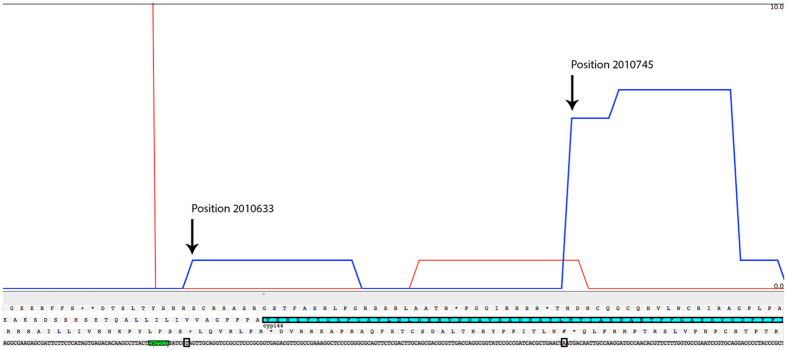
Alternative transcripts of the *CYP144A1* gene produced by *Mtb* H37Rv. The figure shows that two alternative transcriptional start sites (TSS) are located at nucleotides 201063 (CYP144A1-FLV) and 2010745 (CYP144A1-TRV) in the *Mtb* H37Rv genome. These are shown as blue lines in the figure. A higher level of transcription occurs for the *CYP144A1*-TRV version of the gene. TSS are demarcated by black rectangles and show detailed views of the regions around the TSS for the two forms of the gene, identifying the relevant regulatory regions and the start codons (Val1 and Met31, respectively) for CYP144A1-FLV (VRRSPK…) and CYP144A1-TRV (…MTIAKD…).

Analysis of the transcriptome from *Mtb* H37Rv identified a weak transcription start site (TSS) at genome position 2010633, adjacent to a -10 consensus motif (TATTCT, 2010622-2010627) and 7 bp upstream from the annotated CYP144A1 Val1 start codon in Tuberculist^23^. There is near complete amino acid sequence identity between the predicted 434 amino acid residue *Mtb* H37Rv protein and the orthologues found in *Mtb* CDC1551, *M. canetti* CIPT, *Mtb* T46, and *M. bovis* AF2122/97. In previous studies, we expressed and purified this 434 amino acid full-length form of CYP144A1 (referred to hereafter as CYP144A1-FLV), demonstrating that it bound heme and displayed spectroscopic and ligand-binding properties consistent with a P450^22^. However, the best protein sequence alignments of Mtb CYP144A1 with many other mycobacterial CYP144 family P450 sequences are found using a sequence initiating at an internal residue around Met31. This highlighted the possibility that an alternative, shorter form of the *Mtb* CYP144A1 protein might be produced. Consistent with this model, *Mtb* H37Rv transcriptomics analysis identified a second, stronger TSS at position 2010745. The location of the second TSS would be consistent with the generation of a leaderless transcript with Met31 as the start codon. Interestingly, parallel studies of *CYP144* transcripts from the *Mtb* N145 strains (a Beijing isolate similar to *Mtb* HN878) suggest that this organism favours the upstream TSS (Fig. 2). Leaderless transcripts are common in *Mtb*^33^ and are characterized by the absence of a 5′-untranslated region and a Shine-Dalgarno ribosome binding site. They are considered to comprise up to 25% of the transcripts in *Mtb*, suggesting that “truncated” versions of the numerous proteins *Mtb* encodes might play crucial roles in mycobacterial physiology^34^. TSS mapping thus identified both “full-length” and “truncated” versions of CYP144A1 as potential translation products from alternative transcripts. In light of these novel data, we generated plasmid expression constructs encoding both the full-length (CYP144A1-FLV) 434 amino acid protein and the 404 amino acid truncated version (CYP144A1-TRV) forms of CYP144A1. The expression and purification of the two forms of the CYP144A1 protein enabled comparative studies of their properties, and facilitated the crystallization of the proteins in order to obtain the first structural data for *Mtb* CYP144A1.

### Expression and purification of CYP144A1 in its truncated and full-length forms

**Figure 3:**
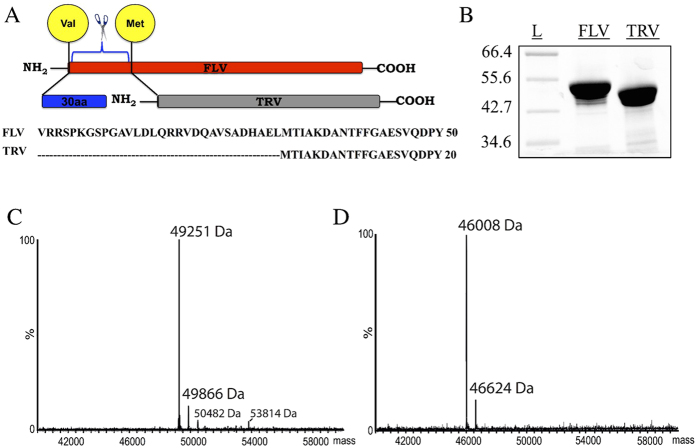
Expression and purification of CYP144A1-FLV and CYP144A1-TRV. Panel (**A**) shows the genetic truncation of the intact CYP144A1-FLV (red) to form CYP144A1-FLV – removing the gene segment encoding residues Va11 to Leu30, shown with the CYP144A1-FLV (Val) and -TRV (Met) initiator residues in yellow circles. This produces the CYP144A1-TRV (grey), with 30 amino acids fewer than CYP144A1-FLV (red). Panel (**B**) shows purification and approximate molecular weight determination of CYP144A1-FLV and -TRV by SDS-PAGE analysis, with markers of indicated mass (kDa) in the first lane. Panels (**C**,**D**) show accurate mass spectra of CYP144A1-FLV (49251 Da) and CYP144A1-TRV (46008 Da) obtained by high-resolution mass spectrometry under denaturing conditions using 40 μM protein in both cases.

The N-terminal truncated version construct of CYP144A1 (CYP144A1-TRV) was generated using the previously prepared full-length version CYP144A1-FLV/pET15b plasmid vector^22^, and by deletion of the region encoding the first 30 amino acids. The CYP144A1-TRV construct initiated from the first methionine codon (Met31) of the *CYP144A1* gene, corresponding to the second of the two CYP144A1 TSS detected (Fig. 3A). CYP144A1-FLV and TRV were both expressed and purified as His_6_-tagged constructs in *E. coli* as described in the *Materials and Methods*. Both the CYP144A1-TRV and CYP144A1-FLV forms were readily purified using nickel affinity, anion exchange and size exclusion column chromatography steps. Both forms of the enzyme were soluble, indicating that the enzyme is almost certainly cytosolic in *Mtb*. This is characteristic for the majority of the *Mtb* P450s characterized to date, including the cholesterol oxidizing CYP125A1 and CYP142A1 enzymes, the branched chain fatty acid hydroxylase CYP124A1 and the cyclodipeptide oxidase CYP121A1^7^,^11^,^13^,^16^,^17^,^18^. A notable exception is CYP128A1, a likely dihydromenaquinone hydroxylase, which may be membrane associated in order to enable access to its lipophilic dihydromenaquinone substrate(s) that are retained in the Mtb cell membrane^14^. The CYP144A1-FLV and CYP144A1-TRV proteins were purified and shown to have approximate masses of 49 kDa and 46 kDa, respectively, as observed by SDS-PAGE analysis (Fig. 3B). The different masses of the two CYP144A1 forms were confirmed using electrospray ionization mass spectrometry, with a mass of 49251 Da determined for CYP144-FLV and 46008 Da for CYP144-TRV (Fig. 3C,D). These values are consistent with the expected masses from the amino acid sequences of the respective forms of the His_6_-tagged CYP144A1 proteins, minus the initial methionine residue (CYP144A1 gene and protein sequences are shown in Supplementary Figure S1), and are also consistent with our previous studies of CYP144-FLV^22^.

### UV-Visible spectroscopic properties of CYP144A1-FLV and CYP144A1-TRV

**Figure 4:**
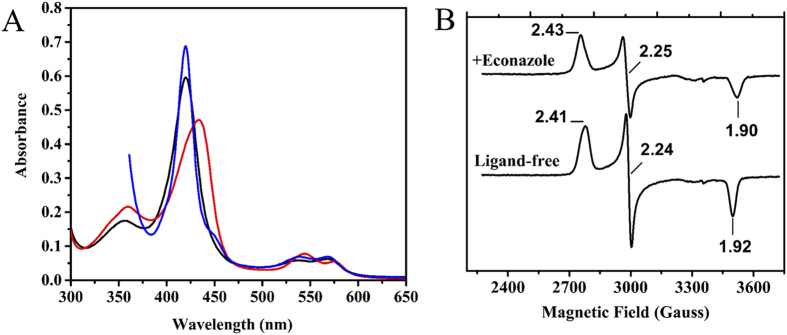
Spectroscopic properties of CYP144A1–TRV. Panel (**A**) UV-visible absorbance spectra of ligand-free CYP144A1-TRV (6 μΜ, black line), nitric oxide-bound (red line) and reduced/carbon monoxide-bound forms (blue line). The Soret spectral maxima are at 420 nm, 437 nm and 420 nm, respectively. Panel (**B**) X-band EPR spectra of ligand-free and econazole (400 μΜ)-bound CYP144A1-TRV (200 μΜ), with the g-values labelled.

UV-Visible (UV-Vis) spectroscopy is a valuable method for characterizing P450s and for the quantitative analysis of their ligand-binding properties. The UV-Vis spectral features of the FLV and TRV forms of CYP144A1 were found to be identical for the two proteins in their oxidized and reduced states, and for these proteins bound to the gaseous ligands nitric oxide (NO, in the ferric form) and carbon monoxide (CO, in the ferrous form). As such, only UV-Vis spectral data for the newly constructed CYP144A1-TRV form are presented (Fig. 4A). The consistency in the spectral properties of the two CYP144A1 proteins suggests that the 30 amino acid N-terminal “extension” on CYP144A1-FLV does not influence the environment of the heme prosthetic group or the coordination of its heme iron. The oxidized form of CYP144A1-TRV has a Soret peak at 420 nm, which is slightly red-shifted in comparison to the resting ferric states of other characterized *Mtb* P450s, such as CYP121A1 at 416.5 nm^13^, CYP51B1 at 419 nm^15^, and CYP130A1 at 418 nm^35^.

The CYP144A1-TRV resting state Soret peak of 420 nm is consistent with that reported previously by Driscoll *et al*.^22^ for CYP144A1-FLV (420.5 nm). Both the FLV and TRV forms of the CYP144A1 enzyme were dialysed extensively (~12 hours) after the nickel-IDA affinity chromatography step of protein purification to ensure the removal of any residual imidazole (used to elute the P450s from the nickel column) that remained bound to the heme iron. The affinity for imidazole is weak (with a *K*_d_ of 3.0 mM for the CYP144A1-FLV form^22^) and so this precautionary step should effect near-complete removal of the ligand. Thus, the 420 nm Soret peak for CYP144A1-TRV almost certainly reflects accurately the properties of the P450 protein in its ferric, low-spin (LS) state, with water as the 6^th^ ligand to the heme iron. The ferric NO-bound CYP144A1-TRV complex showed distinct absorbance properties, with the Soret peak at 437 nm and major spectral changes in the alpha/beta band region with maxima at 576 nm and 544 nm. These features are consistent with those of the NO complexes of the aforementioned *Mtb* P450s CYP51B1, CYP130A1 and CYP121A1^9^,^13^,^15^. The ferrous CO-bound CYP144A1-TRV produced a Soret peak at 420 nm, and thus forms almost completely the cysteine thiol-coordinated (P420) species. Only a minor peak for the cysteine thiolate-coordinated P450 species was detected at 448 nm. The consistency in the UV-Vis spectral properties of CYP144A1-FLV and -TRV in their aqua-ligated ferric, ferric-NO and ferrous-CO complexes provides further evidence that the 30 amino acid N-terminal truncation made to generate the CYP144A1-TRV protein does not alter its heme environment significantly^22^.

### EPR spectroscopic analysis of the CYP144A1-FLV and CYP144A1-TRV proteins

Electron paramagnetic resonance (EPR) X-band spectra were recorded for the oxidized (ferric) CYP144A1-FLV and -TRV proteins to provide further insights into the heme ligation environment and spin-state equilibrium in the two forms of CYP144A1. Spectra for both the FLV and TRV CYP144A1 proteins were similar, with g-values of g_z_ = 2.41, g_y_ = 2.24, and g_x_ = 1.92 (2.41/2.24/1.92) in both cases (Fig. 4B). These data are indicative of a predominantly low-spin ferric and cysteinate-coordinated heme iron. No detectable signals for a ferric high-spin species were detected. The low-spin state of both forms of CYP144A1 is consistent with previous work on the CYP144A1-FLV P450 (g-values of 2.42/2.25/1.93), and these g-values are also similar to those for other *Mtb* P450s in their low-spin states, including the cholesterol hydroxylase CYP142A1 and the cyclodipeptide oxidase CYP121A1^7^,^20^. The EPR spectrum of the CYP144A1-TRV protein bound to the azole drug econazole also shows the complete retention of a low-spin state of the enzyme, but with altered g-values of 2.43/2.25/1.90 (Fig. 4B). Econazole binds tightly to the CYP144A1-FLV protein (*K*_d_ = 0.78 ± 0.29 μM), and an optical titration of CYP144A1-TRV with econazole also revealed a similar *K*_d_ value (0.72 ± 0.18 μM). Econazole binding to CYP144A1-TRV occurs with a heme Soret red shift to 423 nm, which is again similar to that seen for CYP144A1-FLV (Soret A_max_ of the CYP144A1-FLV/econazole complex is at 424 nm). The EPR g-values for the CYP144A1-FLV/econazole complex are at 2.45/2.26/1.89^22^. These data are further confirmatory that the CYP144A1-TRV truncation does not influence the P450 heme environment significantly by comparison with the CYP144A1-FLV enzyme (Fig. 4B).

In the econazole complexes of the CYP144A1 enzyme forms, it might be expected that the g_z_ (and g_x_) values would be more substantially changed from those in the resting, aqua-coordinated state if there was direct coordination of the heme iron by an econazole imidazole nitrogen (e.g. to g_z_ ~2.50 or beyond). Indeed, in previous studies of the CYP144A1-FLV protein a minor g_z_ feature at 2.62 was observed that might indicate direct coordination of the heme iron by an econazole nitrogen in a small proportion of this P450 form^22^. However, the relatively small changes observed in the EPR spectra for the CYP144A1-TRV/FLV proteins may indicate that econazole ligates the heme iron indirectly. This could occur via a retained 6^th^ ligand water molecule in the majority of the P450 molecules for both these proteins. A similar phenomenon was reported for CYP121A1 in complex with fluconazole, and confirmed by X-ray crystallographic data^36^. In this case, the g-values for the CYP121A1-fluconazole complex are 2.45/2.26/1.90, compared to 2.48/2.25/1.90 in the resting form. A similar conclusion can be drawn from the rather less extensive Soret shift of the CYP144A1-FLV/TRV proteins on binding econazole (420.5/420 nm to 424/423 nm) than is observed typically in other P450s (e.g. from 416.5 nm to 423 nm on the binding of econazole to CYP121A1)^20^,^37^.

### Differential Scanning Calorimetry (DSC) studies on the CYP144A1-FLV and CYP144A1-TRV enzyme forms

**Figure 5:**
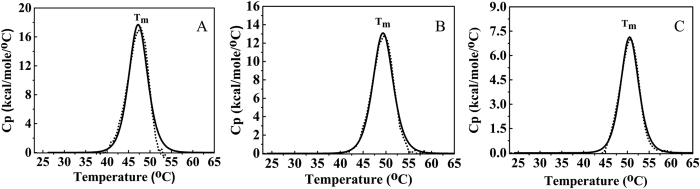
Differential Scanning Calorimetry (DSC) analysis of CYP144A1-TRV. Panels (**A–C**) show DSC thermograms for ligand-free, econazole-bound (30 μΜ), and clotrimazole-bound (30 μΜ) forms of CYP144A1-TRV (8 μΜ), respectively. The collected data (thick black line) and fitting (dotted line) are shown in each case. The T_m_ values are at 47 °C, 50 °C and 50 °C, respectively.

DSC was used to analyse the thermal stability of both the CYP144A1-FLV and -TRV proteins, in order to probe for any differences induced by truncation of the enzyme. The thermal unfolding profiles for both enzymes indicated a single unfolding midpoint temperature (T_m_ value) at 47 °C (Fig. 5A), confirming that the N-terminal truncation did not alter the thermal stability of the CYP144A1-TRV significantly. Further comparative analyses of the thermal stability of the clotrimazole- and econazole-bound forms of CYP144A1 again yielded highly similar results for the CYP144A1-FLV and -TRV proteins. The binding of clotrimazole or econazole increased the T_m_ for both forms of the protein to 50 °C, indicating that the tight-binding azoles stabilize both forms of CYP144A1 to similar extents (Fig. 5B,C). The similarity in thermal stability of both CYP144A1 forms in their ligand-free and azole drug-bound states again indicates that the stability of the CYP144A1-TRV protein is not compromised by the deletion of the 30 amino acid N-terminal segment, and suggests that this part of the protein is not integral to the folding or stability of the structural core of the P450.

### Crystal structure determination of CYP144A1-TRV

**Figure 6:**
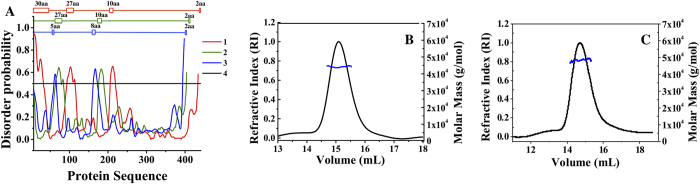
Protein disorder prediction and hydrodynamic features of CYP144A1. Panel (**A**) Protein disorder prediction analysis of CYP144A1-FLV (red), CYP144A1-TRV (green) and the *Mtb* cholesterol hydroxylase CYP142A1 (blue). The threshold (black line) measure of the prediction’s false positive rate was set at 5%. The numbers of disordered amino acids in the protein sequence are indicated on the top panel. The figure was generated using PrDOS software^39^. Panels (**B,C**) Multiangle Laser Light Scattering (MALLS) analysis of CYP144A1-TRV and CYP144A1-FLV, respectively. The refractive index (RI) data are shown as black lines in both cases, with the blue lines indicating the apparent molar mass of the relevant proteins. The data are consistent with both forms of CYP144A1 being monomeric in solution, and having molar masses of ~46,000 g/mol (CYP144A1-TRV) and ~49,000 g/mol (CYP144A1-FLV).

Crystallization of both the FLV and TRV forms of CYP144A1 was achieved, producing crystals with a similar bipyramidal morphology and size (100 μm) in each case. CYP144A1-TRV crystals diffracted to a resolution of 1.55 Å and a ligand-free crystal structure of this form of the CYP144A1 protein was successfully solved using molecular replacement with the *Mtb* cholesterol oxidase enzyme CYP142A1 (PDB 2XKR) as a search model^38^. The CYP144A1-FLV crystals yielded a similar structure (data not shown), with no electron density visible for the additional N-terminal amino acids. This could either reflect that proteolytic cleavage of the N-terminal region of the CYP144A1-FLV form had occurred, or else be due to the fact that the 30 additional N-terminal residues present in CYP144A1-FLV are largely unstructured. Analysis of the CYP144A1-FLV protein sequence using the protein disorder prediction system (PrDOS)^39^ algorithm indicated a disorder probability nearing 90% for these 30 amino acids (Fig. 6A).

**Figure 7:**
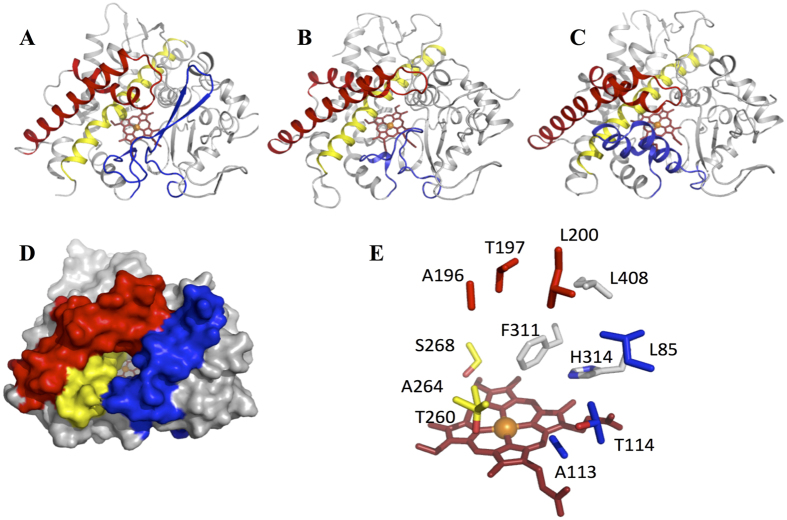
The crystal structure of CYP144A1-TRV. Panels (**A,D**) Ribbon and surface representations, respectively, of the *Mtb* P450 CYP144A1-TRV. The heme, BC-loop, FG-helices and I-helix are coloured red, blue, dark red and yellow, respectively. Panels (**B,C**) Ribbon structure representations of *Mycobacterium smegmatis* CYP142A2 (PDB 2YOO) and the putative orsellinic acid oxidase P450 CalO2 (PDB 3BUJ) using similar colour coding as panel (**A**). The orientation of both structures is similar to that of CYP144A1-TRV in panel (**A**). Panel (**E)** Active site structure of CYP144A1-TRV, with key residues colour coded as in panel (**A**). Panels (**A**–**E**) were generated using PyMOL^48^.

The CYP144A1-TRV crystal structure contains two monomers in the asymmetric unit. However, a dimeric solution state is unlikely, since solution state MALLS experiments (Fig. 6B,C) indicated that both the FLV and TRV forms of CYP144A1 are soluble, monomeric proteins (as are the majority of bacterial P450s). Both monomers in the crystal asymmetric unit have a similar overall conformation, with an rmsd of 0.55 Å for 393 C alpha atoms. The three dimensional structure and secondary structural organization of CYP144A1-TRV resembles those of previously solved *Mtb* P450 structures, and is most similar to P450 CalO2 (PDB 3BUJ), a P450 involved in biosynthesis of the enediyne antitumor antibiotic calicheamicin^40^ (Z score 16.1, rmsd 1.96 Å for 346 C alpha atoms), and to the *Mtb* cholesterol hydroxylase CYP142A1 (PBD code 2YOO) (Z score 15.9, rmsd 1.93 Å for 356 C alpha atoms) (Fig. 7B,C)^7^,^38^.

The structural elements (F/G-helices and BC-loop region) important in determining active site access and substrate specificity^41^ are those that are most distinct in CYP144-TRV from other P450 structures (Fig. 7A). The relatively long linker region connecting the B-and C-helices folds into an extended beta-hairpin, that occupies the region between the N-terminal beta-sheet and the FG-loop. This creates a large access channel to the heme cavity (Fig. 7D), approximately 10 Å deep from the protein surface and 10 Å wide. This suggests the binding of relatively large substrates in CYP144A1.

The active site of CYP144A1-TRV is predominantly hydrophobic, with Phe321 and His324 side chains located in close proximity of the heme 6^th^ ligand. These residues are likely to be important in determining substrate selectivity (Fig. 7E). I-helix residues Glu277 and Ser268 are probably required for protonation of P450 heme iron-oxo species during catalysis. While CYP121A1 also has a serine residue corresponding to Ser268 in CYP144A1-TRV, other *Mtb* P450s use a threonine^7^,^9^,^11^,^15^,^16^,^17^. However, there are few other similarities in the amino acid composition of the active site of CYP144A1 compared to those of the other structurally characterized *Mtb* P450s, suggesting a unique role and substrate selectivity profile for CYP144A1. The substantial structural differences between the active site organization in CYP144A1 and those of the other *Mtb* P450 isoform structures should enable the design of CYP144A1 isoform-selective inhibitors, a topic addressed in our ongoing fragment screening studies of this enzyme.

## Conclusions

There is growing recognition that alternative transcriptional start sites and leaderless transcripts play an important role in increasing the diversity of proteins produced from microbial genomes, with around 25% of mycobacterial transcripts being leaderless^34^. In the case of the *M. tuberculosis* cytochrome P450 CYP144A1, annotation of the relevant P450 (*CYP*) gene in the Tuberculist database indicates that the encoded protein has 434 amino acids with a valine as the first residue (from a GTG codon). Previous studies indicated that this full length form of CYP144A1 (CYP144A1-FLV) could be purified using an *E. coli* expression system^22^. However, a methionine is also located at residue 31 in the CYP144A1-FLV form of the P450, and this was considered as an alternative initiation codon for the protein, particularly in light of the predicted “truncated version” of CYP144A1 (CYP144A1-TRV) being only 404 amino acids in length – a size closer to the norm for a typical prokaryotic P450^6^. Further studies from transcriptomics analysis resulted in the identification of an internal transcriptional start site from a leaderless transcript producing the CYP144A1-TRV form. Both the FLV and TRV forms of the CYP144A1 protein were successfully expressed and purified to homogeneity. Biophysical and biochemical studies revealed similar properties for the two proteins forms, confirming that the 30 N-terminal amino acid residues that were removed in the CYP14A1-TRV form did not affect protein stability, oligomerization state or heme environment. However, bioinformatics studies indicated that the N-terminal 30 amino acid region of CYP144A1-FLV was mainly disordered and that this might explain problems associated with the structural resolution of this region in the crystal structure of the CYP144A1-FLV form of the P450. Consistent with this conclusion, it was found that the CYP144A1-TRV form crystallized readily, allowing the determination of a high resolution (1.55 Å) X-ray structure of the ligand-free CYP144A1-TRV enzyme. This crystal structure shows a P450 with a large and predominantly hydrophobic active site, suggesting that CYP144A1’s natural substrate(s) are bulky hydrophobic molecules. These data provide the basis for biochemical and modelling studies to identify substrates and other ligands for the P450. In this respect, our ongoing work involves fragment based screening studies to identify novel, specific ligands for CYP144A1 that can be developed into useful reagents for use as inhibitors and as mechanistic probes of this *Mtb* P450.

## Methods

### Bioinformatics studies

All protein sequence alignments were performed using the Phylogeny web tool and the NCBI BLAST program^42^,^43^. The individual protein sequences for the CYP144A1-FLV and CYP144A1-TRV P450s were interrogated on the database in searches for sequence neighbours. The resulting output sequences for the various species were further aligned and saved in CLUSTALW format. The phylogenetic tree was generated using MEGA6 software^44^. The aligned CLUSTALW format sequences were imported into the MEGA6 phylogeny tool and a phylogenetic tree was generated using the “construct/test maximum likelihood” method. Structural alignments were performed using the ESPript web tool^45^.

### Identification of CYP144A1 transcriptional start sites

Transcriptional start sites (TSSs) for CYP144A1 were mapped at single-base resolution by sequencing of a preparation of RNA from an exponential culture of *M. tuberculosis* H37Rv after enrichment for transcripts with intact 5′ triphosphate ends, as described by Cortes *et al*.^33^.

### Cloning of the CYP144A1-FLV and TRV genes

The pCYP144A1-FLV construct encoding the full-length (434 amino acid) recombinant *Mtb* CYP144A1-FLV enzyme was produced as previously described^22^. The truncated *Rv1777* gene encoding CYP144A1-TRV was generated by PCR from a previously cloned construct of the full length *Rv1777* gene in the pET15b plasmid vector using the forward primer, 5′-CGATCACGCTGAACAT**ATG**ACAATTGCC-3′ and the reverse primer, 5′-GGCAATTGTCATATGTTCAGCGTGATCG-3′ (Merck-Millipore, Watford UK). The underlined letters in both the forward and reverse primers indicates an engineered NdeI restriction endonuclease site. The bold letters in the forward primer indicate the start codon ATG. The PCR amplification reaction was carried out in a Techne TC-512 thermal cycler (Techne, Cambridge UK) using the proofreading Pfu Turbo DNA polymerase (Agilent, Cheadle UK). The amplification conditions were 95 °C for 2 min, 20 cycles of 95 °C for 30 s, 60 °C for 30 s, and 68 °C for 7 min. The PCR reaction was followed by a final polymerization step of 68 °C for 7 min. The generation of the truncated gene encoding CYP144A1-TRV was achieved by removing the DNA segment between the two NdeI restriction sites through a NdeI endonuclease digest reaction, followed by re-circularization carried out using a Quick Ligation Kit (NEB, Hitchin UK) to produce the pCYP144A1-TRV construct. The resulting CYP144A1-TRV gene construct encodes the CYP144A1-TRV protein with the initial 30 amino acids from the intact CYP144A1-FLV protein deleted.

### Expression and purification of the CYP144A1-FLV and CYP144A1-TRV forms of CYP144A1

CYP144A1-FLV and CYP144A1-TRV proteins were produced by transforming the *E. coli* strain C41 (DE3) (Merck-Millipore, Watford UK) with the pCYP144A1-FLV or pCYP144A1-TRV plasmid constructs. Expression of the relevant gene constructs was done using the isopropyl β-D-thiogalactopyranoside (IPTG)-inducible T7 RNA polymerase/promoter system. This was achieved through IPTG-dependent expression of the T7 RNA polymerase from a chromosomally integrated gene copy in the C41 (DE3) strain, leading to T7 polymerase-dependent transcription of the CYP144A1-FLV and -TRV genes in pET15b. CYP144A1 protein production was typically done in ~15 litre cultures of 2xYT growth medium (ForMedium, Hunstanton UK). The culture medium was distributed between 24 × 2 litre conical flasks. Each flask contained 600 ml of growth medium and ampicillin (50 μg/ml), and was inoculated with 6 ml of transformant cells from an overnight culture in the same medium under the same conditions. Cells were grown at 37 °C with agitation (200 rpm) until an OD_600_ of 0.5 was reached, and then the growth temperature was decreased to 22 °C and bacterial cell growth was continued to an OD_600_ of 0.7. 100 μM IPTG was then added to induce target gene expression, along with 100 μM delta-aminolevulinic acid (ΔALA) to promote heme synthesis and incorporation into the proteins. The transformant cells were then grown for a further 36 h. The cells were harvested by centrifugation at 6000 g for 10 min at 4 °C using a JLA-8.100 rotor in an Avanti J-26 XP centrifuge. The supernatant was discarded and cell pellets were resuspended in ~300 ml of ice cold 50 mM potassium phosphate (KPi, pH 8.0) containing 250 mM NaCl and 10% glycerol. The protease inhibitors phenylmethanesulfonyl fluoride (PMSF, 1 mM), benzamidine hydrochloride (1 mM) and six cOmplete EDTA-free tablets (Roche Diagnostics Ltd, West Sussex UK) were added to inhibit protease enzymes. The cells were lysed on ice by ultrasonication (Bandelin Sonopuls sonicator) with 6 cycles of 30 s on and 60 s rest periods. The cell lysate was centrifuged at 40,000 g for 45 min at 4 °C and the supernatant collected.

The supernatant was loaded onto a Ni-IDA column (Generon, Maidenhead UK) pre-equilibrated with 50 mM KPi (pH 8.0) loading buffer containing 250 mM NaCl and 10% glycerol, using a peristaltic pump (GE Healthcare, Little Chalfont UK). The column was washed with ~80 ml of loading buffer and the flow-through discarded. Proteins were eluted from the column by washing consecutively with increasing concentrations of imidazole [10 mM (250 ml), 80 mM (150 ml) and 160 mM (100 ml) in the loading buffer]. Each eluted fraction was analysed spectrally (250–800 nm), and by SDS-PAGE. Fractions containing relatively pure (mainly the 80 mM and 160 mM) CYP144A1 samples were pooled and concentrated to ~100 ml using ultrafiltration with Amicon concentrators (Merck-Millipore) at 4 °C. The concentrated protein was further dialysed into 50 mM Tris HCl (pH 7.2, dialysis buffer) containing 50 mM KCl and 1 mM EDTA to remove excess imidazole. The dialysed protein was then loaded onto a Q-Sepharose column (10 cm × 4 cm) pre-equilibrated with the dialysis buffer, and then washed and eluted with linear gradient of KCl (50–500 mM) in the dialysis buffer using an automated AKTA purification system (GE Healthcare). Fractions were analysed both spectrally and by SDS-PAGE as before. Samples with high A_420_/A_280_ (or Reinheitszahl, Rz) ratios (≥1) were pooled and concentrated to ~200 μl by ultrafiltration using a Centriprep 30 concentrator (Merck-Millipore) at 1500 g. The concentrated protein was further dialysed into 10 mM Tris HCl (pH 7.5) containing 150 mM NaCl. The protein was then subjected to a final purification step via Sephacryl S-200 size exclusion chromatography column using an AKTA purification system. Fractions were again analysed both spectrally and by SDS-PAGE. Fractions with Rz values of ≥1.5 were pooled and concentrated as before, and dialysed into 50 mM Tris.HCl, pH 7.5 containing 50 mM NaCl and 20% glycerol, and stored at −80 °C until use. Purity of the CYP144A1 proteins was assessed by both SDS-PAGE and UV-Vis spectroscopy, with SDS-PAGE samples migrating as a single band at the appropriate molecular weight on SDS-PAGE gels and having an Rz ratio of ≥1.5 being considered as pure.

### Mass spectrometry

Protein solutions for mass spectrometry (40 μM) were prepared by dilution of purified proteins (500–1000 μM) in 200 μM ammonium acetate buffer, pH 7.0. Liquid chromatography-mass spectrometry (LC–MS) was performed on a Xevo G2-S Q-TOF UPLC instrument (Waters, Elstree UK) coupled to an Acquity UPLC system. Samples were eluted through an Acquity UPLC BEH300 C4 column (1.7 μm, 2.1 × 50 mm) using a mobile phase of Solvent A: water with 0.1% formic acid, and Solvent B: 95% acetonitrile containing 0.01% formic acid. The elution gradient was run using 95% Solution A for 5.21 minutes, 100% Solution B for 1 minute, and 100% Solution A for 1 minute at a flow rate of 0.2 ml min^−1^ over a total run time of 7.29 minutes. The electrospray source was operated with a capillary voltage of 2.0 kV and a cone voltage of 40 V. Nitrogen was used as the desolvation gas at a total flow of 850 litres hr^−1^. Data acquisition and processing was performed using Micromass MassLynx v4.1 software with total mass spectra reconstructed from the ion series using the pre-installed MaxEnt algorithm.

### Determination of molecular weight and aggregation state of CYP144A1-FLV and CYP144A1-TRV by multi-angle laser light scattering (MALLS)

An estimate of the molecular weight of both CYP144A1-FLV and CYP144A1-TRV proteins, as well as analysis of their aggregation states, was obtained using a MALLS detector (Wyatt DAWN Eos, Haverhill UK). This was immediately preceded by passing the sample through a Superdex 200 gel filtration step integrated into the detector (24 ml S200 10/300 GL, GE Healthcare). The column was run at a flow rate of ~0.8 ml/min, using 100 μl samples of 2 mg/ml CYP144A1 enzymes in 10 mM Tris.HCl buffer (pH 7.5) containing 150 mM NaCl. An Optilab rEX and Quasi Elastic Light Scattering (QELS) apparatus (both from Wyatt) were used to obtain the refractive index and the hydrodynamic radius of CYP144A1 enzymes, respectively. A K5 cell type and a laser wavelength of 690 nm were used to collect data at 1 s intervals.

### EPR spectroscopic analysis of CYP144A1

Continuous wave X-band electron paramagnetic resonance (EPR) spectra of CYP144A1 proteins were obtained at 10 K using a Bruker ELEXSYS E500 EPR spectrometer equipped with an ER 4122SHQ Super High Q cavity. Temperature control was effected using an Oxford Instruments ESR900 cryostat connected to an ITC 503 temperature controller. Microwave power was 0.5 mW, modulation frequency was 100 KHz and the modulation amplitude was 5 G. EPR spectra were collected for the CYP144A1-FLV and CYP144A1-TRV proteins in the ligand-free state (200 μM) and at the same protein concentration following the addition of the azole inhibitor drug econazole (400 μM).

### Determination of CYP144A1 thermal stability by Differential Scanning Calorimetry (DSC)

DSC Experiments were performed using a Microcal VP-DSC instrument (Malvern Instruments, Malvern UK). Parameters used for running samples were 20–80 °C temperature gradient, 10 min prescan thermostat and a 90 °C/h scan rate. Baseline scans were performed using assay buffer (10 mM KPi, 100 mM NaCl, pH 7.0) and proteins were near-saturated with the azole ligand clotrimazole and econazole prior to DSC analysis of ligand-bound samples. All samples were run using 8 μM protein and azole ligands were used at concentrations at least 20x those of the protein and according to the affinity for the azoles. Data analysis was performed using Origin Software (OriginLab, Northampton MA).

### CYP144A1 crystallization and structure determination

Crystallization was performed using the sitting drop method using 20 mg/ml CYP144A1. Drops were prepared by the addition of 0.2 μl of CYP144A1-FLV and -TRV proteins to 0.2 μl of mother liquor, and by incubating at 4 °C. Following initial crystallogenesis using commercial screens, crystallization conditions were further refined using the sitting drop vapour diffusion technique to 0.8 or 1.0 M (NH_4_)_2_SO_4_ with 0.1 M HEPES, pH 7.55, and 25% PEG 3350. Single crystals were flash cooled after addition of 10% PEG 200 as cryoprotectant, and data were collected at Diamond synchrotron beamline IO3 (Harwell, UK). The CYP144A1-TRV structure (PDB 5HDI) was solved by molecular replacement with the *M. tuberculosis* CYP142A1 structure (PDB 2XKR) as the template. Following automatic model building and refinement using Buccaneer from the CCP4 suite^46^, the model was completed using iterative rounds of refinement using Refmac5 intercalated with manual model building using COOT^47^. Data collection and refinement parameters for the CYP144-TRV crystal structure are presented in Table 1.

## Additional Information

**How to cite this article**: Chenge, J. *et al*. Structural characterization of CYP144A1 – a cytochrome P450 enzyme expressed from alternative transcripts in *Mycobacterium tuberculosis*. *Sci. Rep*. **6**, 26628; doi: 10.1038/srep26628 (2016).

## Supplementary Material

Supplementary Information

## Figures and Tables

**Table 1:** Data reduction and final structural refinement statistics for *Mycobacterium tuberculosis* CYP144A1.

	CYP144A1 (PDB 5HDI)
**Data collection**	
X-ray Source	Diamond, IO3
Wavelength, Å	1.0
Space group	P2_1_2_1_2_1_
Cell dimensions	
a, b, c (Å)	58.30, 117.78, 122.09
Molecules per asymmetric unit	2
*R*_merge_ (%)	9.7 (80.7)
*I*/σ*I*	10.4 (2.3)
*CC (1/2)*	99.9 (67.5)
Completeness (%)	98.7 (97.1)
Redundancy	4.4 (4.4)
Wilson B factor (Å^2^)	15.7
**Refinement**	
Resolution (Å)	88.58-1.54 (1.58–1.54)
No. reflections	116903 (8401)
*R*_work_/*R*_free_	13.76/19.24 (21.4/28.3)
No. non-hydrogen atoms	6850
Mean B factor (Å^2^)	22.5
R.m.s. deviations	
Bond lengths (Å)	0.019
Bond angles (º)	1.872
Ramachandran plot	
Favourable regions (%)	96.7
Allowed regions (%)	2.9
Outliers (%)	0.4

Data were collected using beamline IO3 at the Diamond synchrotron (Harwell, UK).
