# Supplementary material for: Structural characterization of CYP144A1 – a cytochrome P450 enzyme expressed from alternative transcripts in Mycobacterium tuberculosis
Source: Sci Rep. 2016 May 26;6:26628. doi: 10.1038/srep26628 (PMC4880925; doi:10.1038/srep26628)
Supplement: Supplementary Information [file srep26628-s1.doc]

**SUPPLEMENTARY DATA**

**Structural characterization of CYP144A1 – a cytochrome P450 enzyme expressed from alternative transcripts in *Mycobacterium tuberculosis***

Jude Chenge^1†^, Madeline E. Kavanagh^2†^, Max D. Driscoll^1^, Kirsty J. McLean^1^, Douglas B. Young^3^, Teresa Cortes^4^, Dijana Matak-Vinkovic^2^, Colin W. Levy^1^, Stephen E. J. Rigby^1^, David Leys^1^, Chris Abell^2^, Andrew W. Munro^1*^

^1^Manchester Institute of Biotechnology, Centre for Synthetic Biology of Fine and Specialty Chemicals (SYNBIOCHEM), Faculty of Life Sciences, The University of Manchester, Manchester M1 7DN, United Kingdom. ^2^Department of Chemistry, University of Cambridge, Lensfield Road, Cambridge CB2 1EW, United Kingdom. ^3^Centre for Molecular Microbiology and Infection, Imperial College London, London, United Kingdom. ^4^Department of Pathogen Molecular Biology, Faculty of Infectious and Tropical Diseases, London School of Hygiene and Tropical Medicine, Keppel Street, London WC1E 7HT, United Kingdom.

^*^Corresponding Author

Email: andrew.munro@manchester.ac.uk

Phone: 0044 161 3065151

**Supplementary Dataset 1**. **Gene and protein sequences for the *Mycobacterium tuberculosis* CYP144A1**. The gene/amino acid sequences for the native Mtb CYP144A1 are shown overlaid. Alternative transcripts produce either a 434 amino acid full length version of CYP144A1 (CYP144A1-FLV, starting from gtg/Val1, in red text) or a 404 amino acid truncated version (CYP144A1-TRV, starting from atg/Met31, also in red text). In the pET15b expression constructs used, an N-terminal His_6_-tag DNA sequence is attached to the FLV and TRV versions of the *CYP144A1* gene. The sequence encodes the 20 amino acid oligopeptide GSSHHHHHHS SGLVPRGSHM, with the final methionine being the first amino acid of both forms of the P450 protein (replacing the valine in the case of the CYP144A1-FLV protein).

**gtg** aga cgt tcg ccg aaa ggc tcc ccg ggc gca gtt ctc gac ttg cag cga cgc gtt gac 60

**Val**-Arg-Arg-Ser-Pro-Lys-Gly-Ser-Pro-Gly-Ala-Val-Leu-Asp-Leu-Gln-Arg-Arg-Val-Asp 20

cag gcg gta tcc gcc gat cac gct gaa cta **atg** aca att gcc aag gat gcc aac acg ttc 120

Gln-Ala-Val-Ser-Ala-Asp-His-Ala-Glu-Leu-**Met**-Thr-Ile-Ala-Lys-Asp-Ala-Asn-Thr-Phe 40

ttt ggt gcc gaa tcc gtg cag gac ccc tac ccg ctg tat gag cgc atg cgc gcc gca ggc 180

Phe-Gly-Ala-Glu-Ser-Val-Gln-Asp-Pro-Tyr-Pro-Leu-Tyr-Glu-Arg-Met-Arg-Ala-Ala-Gly 60

tcg gtc cac cgg atc gct aac tcg gac ttc tat gcc gtg tgc ggt tgg gac gct gtc aat 240

Ser-Val-His-Arg-Ile-Ala-Asn-Ser-Asp-Phe-Tyr-Ala-Val-Cys-Gly-Trp-Asp-Ala-Val-Asn 80

gag gcc atc ggt cgt ccg gag gac ttc tcc tcg aat ttg acc gcc acg atg acc tat acg 300

Glu-Ala-Ile-Gly-Arg-Pro-Glu-Asp-Phe-Ser-Ser-Asn-Leu-Thr-Ala-Thr-Met-Thr-Tyr-Thr 100

gcc gag ggc acc gct aaa ccg ttc gag atg gac cca ctc ggc gga ccc aca cac gtg ttg 360

Ala-Glu-Gly-Thr-Ala-Lys-Pro-Phe-Glu-Met-Asp-Pro-Leu-Gly-Gly-Pro-Thr-His-Val-Leu 120

gcc acc gcc gac gat cct gcc cac gcc gtg cac cgc aag ctc gtg ctg cgt cac ttg gcg 420

Ala-Thr-Ala-Asp-Asp-Pro-Ala-His-Ala-Val-His-Arg-Lys-Leu-Val-Leu-Arg-His-Leu-Ala 140

gcc aag cgg atc cgc gtt atg gag cag ttc acc gta cag gct gcc gac cgg ctg tgg gtc 480

Ala-Lys-Arg-Ile-Arg-Val-Met-Glu-Gln-Phe-Thr-Val-Gln-Ala-Ala-Asp-Arg-Leu-Trp-Val 160

gac ggc atg cag gat ggg tgc atc gaa tgg atg ggc gcc atg gcc aat cgc cta ccg atg 540

Asp-Gly-Met-Gln-Asp-Gly-Cys-Ile-Glu-Trp-Met-Gly-Ala-Met-Ala-Asn-Arg-Leu-Pro-Met 180

atg gtc gta gct gag ctc atc ggc ctg ccc gac ccc gac atc gcc cag ctg gtg aag tgg 600

Met-Val-Val-Ala-Glu-Leu-Ile-Glu-Leu-Pro-Asp-Pro-Asp-Ile-Ala-Gln-Leu-Val-Lys-Trp 200

gga tac gcg gcc act cag cta ctc gaa ggg ttg gtc gaa aac gat cag ctc gtc gcc gcg 660

Gly-Tyr-Ala-Ala-Thr-Gln-Leu-Leu-Glu-Gly-Leu-Val-Glu-Asn-Asp-Gln-Leu-Val-Ala-Ala 220

ggt gtg gcg ttg atg gag ctc agc ggt tac atc ttc gag cag ttt gac cgt gcc gcg gcc 720

Gly-Val-Ala-Leu-Met-Glu-Leu-Ser-Gly-Tyr-Ile-Phe-Glu-Gln-Phe-Asp-Arg-Ala-Ala-Ala 240

gat ccg cgg gac aat ctg ctc ggt gag ctt gcc acc gcc tgc gca tcg ggg gag ctg gac 780

Asp-Pro-Arg-Asp-Asn-Leu-Leu-Gly-Glu-Leu-Ala-Thr-Ala-Cys-Ala-Ser-Gly-Glu-Leu-Asp 260

act ctc acc gcc cag gtc atg atg gtc acc ttg ttc gcc gcc ggc ggc gag tcc acg gcg 840

Thr-Leu-Thr-Ala-Gln-Val-Met-Met-Val-Thr-Leu-Phe-Ala-Ala-Gly-Gly-Glu-Ser-Thr-Ala 280

gcg ctg ctg ggc agc gcg gta tgg ata ctg gcg aca cgt ccc gat atc cag caa cag gtg 900

Ala-Leu-Leu-Gly-ser-Ala-Val-Trp-Ile-Leu-Ala-Thr-Arg-Pro-Asp-Ile-Gln-Gln-Gln-Val 300

cgc gcg aac ccc gag ctg ctg gga gcg ttt atc gaa gag acg ctg cgt tac gag ccg cca 960

Arg-Ala-Asn-Pro-Glu-Leu-Leu-Gly-Ala-Phe-Ile-Glu-Glu-Thr-Leu-Arg-Tyr-Glu-Pro-Pro 320

ttt cgc ggc cac tac cgc cac gtg cga aac gcc acc acc ttg gac ggc acg gaa ctg ccc 1020

Phe-Arg-Gly-His-Tyr-Arg-His-Val-Arg-Asn-Ala-Thr-Thr-Leu-Asp-Gly-Thr-Glu-Leu-Pro 340

gcg gat tcg cac ctg ctg ctg ttg tgg ggc gcg gcc aac cgc gat cca gcc cag ttc gag 1080

Ala-Asp-Ser-His-Leu-Leu-Leu-Leu-Trp-Gly-Ala-Ala-Asn-Arg-Asp-Pro-Ala-Gln-Phe-Glu 360

gca ccc ggc gag ttc cgt ctt gac cgt gca gga ggc aaa ggc cac atc agt ttc gga aaa 1140

Ala-Pro-Gly-Glu-Phe-Arg-Leu-Asp-Arg-Ala-Gly-Gly-Lys-Gly-His-Ile-Ser-Phe-Gly-Lys 380

ggg gcc cac ttc tgt gtc ggc gct gca ctg gca cgc ttg gag gct cga atc gtc ttg cgt 1200

Gly-Ala-His-Phe-Cys-Val-Gly-Ala-Ala-Leu-Ala-Arg-Leu-Glu-Ala-Arg-Ile-Val-Leu-Arg 400

ctg ctg ctc gat cgc acc tcg gta att gag gca gcc gat gtc ggc ggg tgg ttg ccc agt 1260

Leu-Leu-Leu-Asp-Arg-Thr-Ser-Val-Ile-Glu-Ala-Ala-Asp-Val-Gly-Gly-Trp-Leu-Pro-Ser 420

atc ctg gtg cgc cgc atc gag cgg cta gag cta gct gta caa tag 1305

Ile-Leu-Val-Arg-Arg-Ile-Glu-Arg-Leu-Glu-Leu-Ala-Val-Gln 404/434
